# Gut Microbiota in Colorectal Cancer: Mechanisms of Carcinogenesis, Biomarkers, and Therapeutic Perspectives

**DOI:** 10.3390/jcm15145331

**Published:** 2026-07-08

**Authors:** Bianca Andreea Cristinescu, Horatiu Dura, Sorin Radu Fleaca, Danusia Maria Onisor, Olga Brusnic, Corina Porr, Sabrina Birsan, Adrian Boicean

**Affiliations:** 1 Medical Clinical Department, Faculty of Medicine, “Lucian Blaga” University, 550024 Sibiu, Romania; horatiu.dura@ulbsibiu.ro (H.D.); radu.fleaca@ulbsibiu.ro (S.R.F.); corina_sibiu@yahoo.com (C.P.); sabrinaandreea.marinca@ulbsibiu.ro (S.B.); adrian.boicean@ulbsibiu.ro (A.B.); 2 County Clinical Emergency Hospital of Sibiu, 550245 Sibiu, Romania; 3 Department of Internal Medicine VII, George Emil Palade University of Medicine, Pharmacy, Science and Technology of Targu Mures, 540136 Targu Mures, Romania; halalisan5@yahoo.com (D.M.O.); brusnic_olga@yahoo.com (O.B.)

**Keywords:** gut microbiota, metabolites, biomarkers, dysbiosis, colorectal cancer

## Abstract

Colorectal cancer (CRC) remains one of the leading causes of cancer-related mortality worldwide, largely due to late-stage diagnosis and the limited sensitivity of current screening approaches for early lesions. In recent years, the gut microbiota has emerged as a key factor in colorectal carcinogenesis, offering promising opportunities for the development of novel, non-invasive diagnostic and therapeutic strategies. Specific bacterial species and microbial metabolites have been implicated in colorectal carcinogenesis and are under investigation as potential biomarkers for CRC detection. However, despite growing evidence of association, most remain investigational and require further clinical validation before routine implementation. Among the proposed bacterial biomarkers, *Fusobacterium nucleatum* is one of the most promising candidates for clinical implementation, with encouraging evidence supporting its use in non-invasive stool-based CRC detection. *Streptococcus gallolyticus* subsp. *gallolyticus* and *Clostridium septicum* currently serve primarily as clinical warning markers due to their well-established association with occult colorectal malignancy rather than as screening biomarkers. In contrast, pks^+^ *Escherichia coli*, enterotoxigenic *Bacteroides fragilis*, *Enterococcus faecalis*, and *Peptostreptococcus anaerobius* remain investigational. *Helicobacter pylori* has limited value for CRC detection because of its inconsistent association and low specificity. Overall, this review summarizes the most extensively studied bacterial species and microbial metabolites involved in colorectal cancer development, with particular emphasis on their underlying pathogenic mechanisms and highlighting their potential value as diagnostic biomarkers and therapeutic targets. It also highlights emerging bacterial taxa and microbial signatures that have been associated with early tumorigenesis in recent studies.

## 1. Introduction

Colorectal cancer ranks third in terms of incidence worldwide, being the second leading cause of cancer-related mortality [1]. The highest age-standardized incidence rates are observed in developed countries/continents like Australia, New Zealand, the United States of America, Canada, Europe, and Japan. In 2022, Australia ranked first in colorectal cancer (CRC) incidence, with a rate of 462.5 per 100,000 population [1,2]. In contrast, developing countries exhibit the lowest rate of CRC. This disparity may reflect variations in lifestyle and dietary habits or underdiagnosis, given the fact that countries undergoing major lifestyle and dietary transitions are experiencing steadily increasing incidence rates. Even though the incidence and mortality slightly decreased in high-income countries, CRC remains a leading cause of cancer-related morbidity and mortality worldwide, largely due to late-stage diagnosis and limitations of current screening methods [1,2].

**Figure 1:**
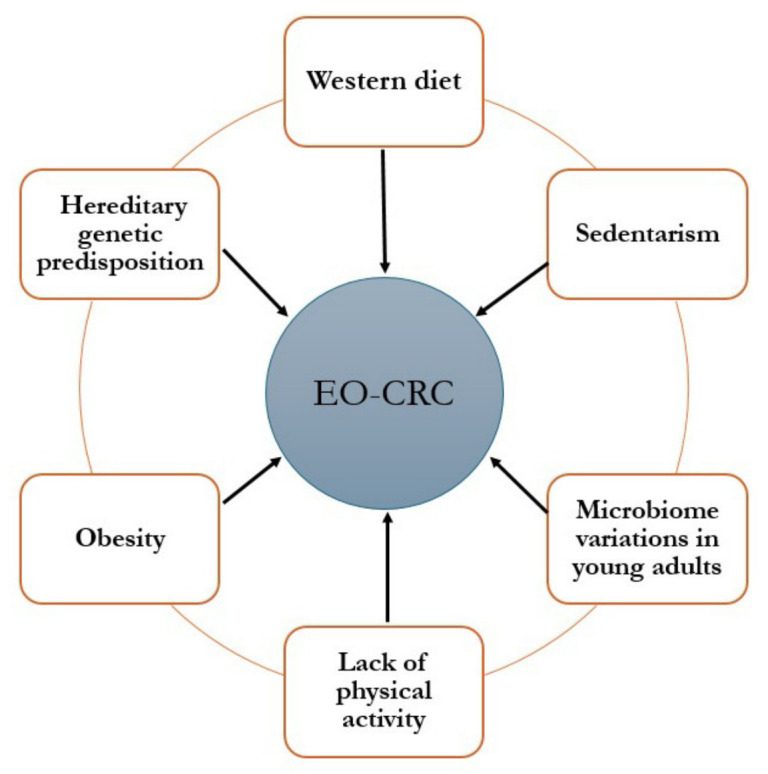
The factors that contribute to Early Onset of Colorectal Cancer. Abbreviation: Early Onset Colorectal Cancer, EO-CRC.

The new arising concern is that there is a trend toward an increased occurrence of CRC at a younger age (<50 years), called early onset CRC (EO-CRC) [3]. The main factors contributing to this are thought to be the Western diet, sedentarism, lack of physical activities, and obesity, correlated with microbiome variations in younger adults [3,4]. Up to 25% of EOCRC cases are associated with germline pathogenic variants, supporting recommendations for universal germline multigene panel testing to improve diagnosis, guide treatment, and enable family screening. However, the majority of EOCRC cases are sporadic and lack identifiable hereditary predisposition. These sporadic tumors often present with more aggressive clinical features, and their rising incidence is thought to be driven by environmental and lifestyle factors (Figure 1) [5].

The gut microbiota is highly variable and can be influenced by age, sex, gender, geographical location, ethnicity, genetics (familial adenomatous polyposis, Lynch syndrome), mode of delivery, exposure to breastfeeding, smoking, alcohol consumption, physical activity, stress, type of diet, obesity, use of medication (antibiotics), early exposure to antibiotics, disease, and comorbidities [6,7]. Epidemiological data indicate that males have a higher overall incidence of CRC compared with females. However, women are more frequently diagnosed with aggressive right-sided colon cancers [8].

Unhealthy dietary patterns, particularly the Western diet, characterized by high consumption of red and processed meats, refined sugars, and sodium, alongside low intake of fiber-rich foods, have been consistently associated with an increased risk of colorectal cancer. Red and processed meat form genotoxic and *N*-nitroso compounds (NOCs) with carcinogenic effects. Heme iron found abundantly in meat promotes colonocyte proliferation; trimethylamine-*N*-oxide (TMAO) promotes inflammation, while dietary fat increases the production of secondary bile acids and oxidative DNA damage [8,9]. Higher sugar intake, especially sweetened beverages, alters the insulin-like growth factor (IGF-1) axis, which leads to insulin resistance and inflammation [10,11]. Excessive dietary salt intake has been associated with gut microbiota alterations and enhanced pro-inflammatory immune responses, including T helper 17 activation and interleukin-17 (IL-17) production. [12].

On the other hand, consumption of fruits, vegetables, fish, fiber, whole grains, dairy, and non-steroidal anti-inflammatory drugs has a protective role in CRC development. Fruits and vegetables are rich in flavonoids, which have an anti-inflammatory and antioxidant effect by modulating immune cell function and protecting the gut barrier. High dairy consumption has been associated with a reduced risk of colorectal cancer incidence and mortality, potentially attributable to the protective effects of calcium, vitamin D, lactoferrin, and butyric acid [8,13,14,15]. Omega-3 found in fish oil reduces inflammation-promoting bacteria (Enterobacteriaceae and Clostridium) and increases beneficial bacteria like Akkermansia, Lactobacillus, and Bifidobacterium, thereby reducing bacterial overgrowth and suppressing inflammation [16]. Fibers, through fermentation, produce SCFAs such as butyrate, acetate, and propionate. SCFAs exert their effects through G protein-coupled receptor signaling and histone deacetylase inhibition by promoting immunosuppressive effects and anti-inflammatory pathways [17]. The interaction between butyrate and dietary fat was demonstrated by Hong et al. in an in vivo study on rats, in which the combination of butyrate and fish oil exerted a protective effect against CRC tumorigenesis. This association promoted apoptosis and reduced cellular proliferation compared with the combination of butyrate and corn oil. Notably, the pro-apoptotic effect of butyrate was observed only when administered in conjunction with fish oil, whereas its combination with corn oil abolished the apoptotic response [18].

Obesity increases the risk of CRC through increased cell proliferation, inhibition of tumor cell apoptosis, and altered gene expression. Obesity-associated gut dysbiosis exhibits alterations of diversity, richness, and composition leading to increased secondary bile acids and a decrease in SCFAs. Adipose tissue causes inflammation by secreting adipokines and various cytokines like interleukin-1, interleukin-6 (IL-6), IL-17, interleukin-32α, tumor necrosis factor-α (TNF-α), etc., leading to activation of nuclear factor kappa B (NF-κB), c-Jun N-terminal kinase (JNK), and protein kinase R pathways. The concomitant increase in leptin and decrease in adiponectin establish a state of low-grade inflammation. Furthermore, obesity-driven insulin resistance and alterations in the IGF-1 signaling pathway enhance cell proliferation while suppressing apoptotic mechanisms [19,20,21,22].

## 2. Materials and Methods

We conducted comprehensive literature research across Web of Science (WoS) and PubMed using keywords such as “gut microbiota”, “colorectal cancer”, “carcinogenesis” and “bacteria”, initially retrieving 475 articles in WoS and 613 articles in PubMed. We limited the search to papers published between 2020 and 2026 (320-WoS, 419-PubMed), without leaving out the most important papers from before 2020—29 articles. By selecting only relevant research categories (Gastroenterology, Oncology, and Microbiology, etc.), we narrowed the search to 264 papers—WoS and 419 papers—PubMed. We then eliminated duplicate and non-relevant articles. The exclusion criteria comprised papers unrelated to the process of colorectal tumorigenesis caused by the gut microbiome, as well as studies focused on oral or tissue microbiota. After a detailed screening process, 147 published articles were included in the review.

**Figure 2:**
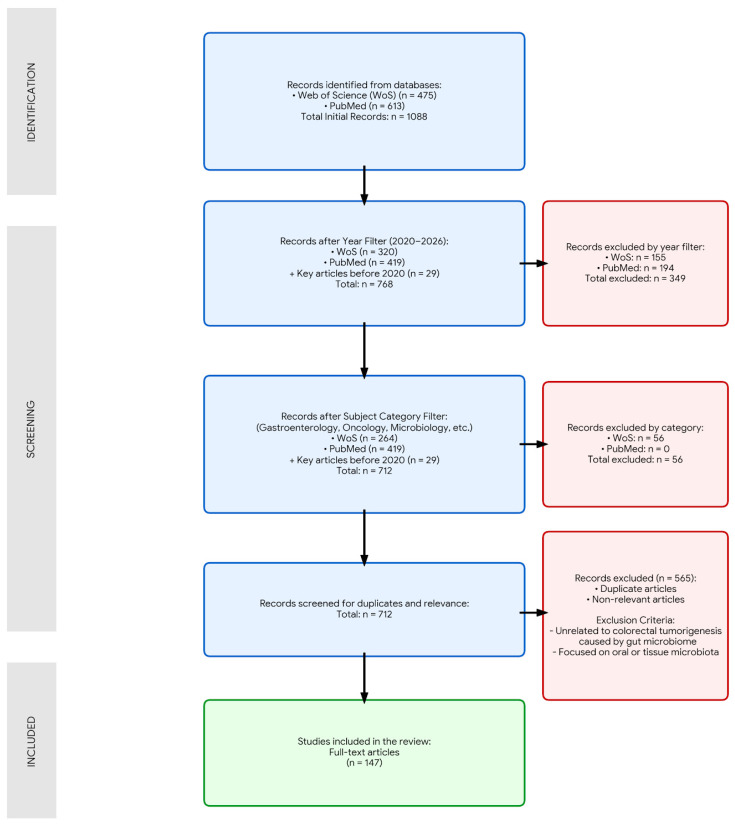
PRISMA flow diagram of the article selection process. Abbreviation: WoS-Web of Science.

In response to reviewer suggestions, additional peer-reviewed studies were incorporated to expand the discussion on sporadic versus IBD-associated colorectal carcinogenesis. These studies were not identified through the original PRISMA-based search strategy but were included to ensure adequate coverage of key mechanistic concepts and to address the requested topic in sufficient depth (Figure 2).

## 3. Gut Microbiota and Colorectal Cancer Pathogenesis

### 3.1. The Driver-Passenger and Alpha Bugs Hypotheses in CRC

Numerous factors contribute to the development of colorectal cancer. It has been well established that the gut microbiota has an essential role in carcinogenesis. Two hypothesis-driven models have been proposed to describe the sequence of tumor development. Sears and Pardoll brought the concept of “alpha bugs”. These are described as bacteria with unique virulence that induce inflammation, cell proliferation, and production of genotoxin substrates and can reshape the microbiota to support the mucosal-induced responses and changes leading to colorectal cancer [23].

Tjalsma et al. proposed a “bacterial driver-passenger” model, in which there are specific driver bacteria like *Streptococcus gallolyticus* (*S. gallolyticus*), enterotoxigenic *Bacteroides fragilis* (*ETBF*), *Enterococcus faecalis* (*E. faecalis*), and *Escherichia coli* (*E. coli*) that initiate the tumorigenesis process by producing reactive oxygen species (ROS), extracellular superoxide, and toxins such as colibactin through signal transducer and activator of transcription 3 (STAT3), E-cadherin, and tumor growth factor-β (TGF-β) pathways. After the adenoma-carcinoma progression, the driver bacteria are replaced by opportunistic (passenger) bacteria that facilitate disease progression. The passenger bacteria, Fusobacterium *nucleatum* (*F. nucleatum*) and *S. gallolyticus,* slowly replace the initial bacteria because of better adaptation to the tumor environment [23,24,25,26,27,28]. The driver-passenger and alpha-bug models provide useful frameworks for understanding host-microbe interactions in colorectal carcinogenesis; however, definitive evidence establishing specific bacteria as primary causal initiators in human CRC remains limited.

### 3.2. The Role of Gut Microbial Metabolites in CRC Development

The gut microbiota generates a variety of metabolites capable of influencing host homeostasis in both beneficial and harmful ways. SCFAs, especially butyrate, have a clear role in protecting against CRC development, whereas deoxycholic acids, lithocholic acids, hydrogen sulfide, and nitroso compounds are considered harmful. TMAO has potential pro-inflammatory and pro-tumorigenic effects, although its causal role remains uncertain and clinical findings are inconsistent, while 3-oxo-lithocholic acid (3-oxo-LCA) exhibits immunomodulatory properties that may contribute to anti-inflammatory pathways. Some tryptophan metabolites, notably indoles, have an anti-inflammatory effect, while others promote inflammation. Polyamines have a dual role in tumorigenesis, depending on the quantity. Ursodeoxycholic acid (UDCA) has not been sufficiently studied, as ambiguous results exist [29,30,31].

#### 3.2.1. SCFAs

The main SCFAs produced by the gut through fermentation are butyrate, acetate, and propionate. Butyrate is a histone deacetylase inhibitor produced by fermentation of dietary fibers and plays the most important role, exerting anticancer properties by modulating the immune system and maintaining the integrity of the intestinal barrier. Being the main source of energy for healthy colonocytes, it usually promotes cell differentiation and growth. However, in CRC cells, the Warburg effect is altered, thereby butyrate accumulation promotes cell apoptosis and suppresses cancer cell proliferation and invasion [16,32,33,34].

Acetate, the most abundant SCFA, regulates the hypothalamic responses, thus the appetite, and modulates lipid metabolism. Propionate has anti-inflammatory properties, induces cell apoptosis, and inhibits cholesterol synthesis [35,36].

#### 3.2.2. Secondary Bile Acids

The main secondary bile acids are deoxycholic acid (DCA) and lithocholic acid (LCA). DCA triggers inflammation by activating the NF-κB pathway and induces apoptosis in a p53-independent way by causing DNA damage to colonic cells, thus disrupting epithelial integrity. It further activates the Takeda G protein bile acid receptor 5 and interferes with the Farnesoid X receptor (FXR), inducing leucine-rich repeat-containing G-protein coupled receptor 5 + cell growth and proliferation [33,37]. LCA uses similar pathways to DCA in enhancing tumor promotion in CRC, mainly by activating extracellular signal-regulated kinase (ERK1/2) and inhibiting STAT3 [38].

High-fat and low-fiber diets, such as Western diets, increase the production of secondary bile acids, promoting CRC development. On the other hand, low-fat and high-fiber diets decrease the incidence of colorectal cancer [39].

#### 3.2.3. Tertiary Bile Acids

Though structurally derived from secondary bile acids (and often referred to as such in recent immunological literature) [40], UDCA and 3-oxo-LCA are sometimes distinguished as tertiary bile acids to reflect the fact that they are not direct products of primary bile acid dehydroxylation, but rather the result of subsequent, multi-step microbial epimerization or oxidation of the secondary pool [41].

UDCA is generated from chenodeoxycholic acid through the activity of bacterial hydroxysteroid dehydrogenases and is a hydrophilic bile acid, unlike DCA and LCA, which are more hydrophobic. There is currently ambiguous and sometimes conflicting evidence regarding the biological effects of UDCA in CRC. While several studies attribute anti-inflammatory and cytoprotective properties to UDCA, other reports suggest that, in the presence of specific gut bacteria, including *Clostridium scindens* and *Clostridium hiranonis*, UDCA may undergo further biotransformation into LCA. Therefore, further studies are required to elucidate the conditions under which UDCA may exert either protective or potentially deleterious effects [40,42].

3-oxo-LCA is an FXR agonist derived from LCA via specific bacteria, like *Eggerthella lenta*. In various mouse models, 3-oxo-LCA reduced bile acid levels, enhanced gut barrier function, promoted tumor apoptosis, and suppressed CRC initiation and progression. These findings highlight the complex interaction between microbial BAs and the FXR pathway, positioning 3-oxo-LCA as a promising candidate for FXR-targeted CRC therapies [41].

#### 3.2.4. Hydrogen Sulfide (H_2_S)

Hydrogen sulfide is considered a tumor-promoting metabolite in colorectal cancer. It is produced endogenously by certain sulfate-reducing bacteria. In a balanced gut microbiota, more than 90% of H_2_S is metabolized and utilized locally within the colon. Although some rodent studies suggest that H_2_S may exert protective effects in certain pathological contexts, its impact on colonocytes appears to be more complex. Elevated concentrations of H_2_S have been associated with genotoxic effects, and sustained exposure to high luminal H_2_S levels has been shown to promote epithelial hyperproliferation [43,44].

#### 3.2.5. N-Nitroso Compounds

NOCs are a class of chemical compounds that induce DNA damage. They are produced by combining nitrites and amines or amides and are strongly related to processed red meat consumption, as the heme iron promotes the reduction in nitrates to nitrites. Another source of nitrites is derived from the metabolic activity of certain intestinal bacteria, including wild-type and mutant strains of *E. coli* as well as various species of Clostridium. These microorganisms possess nitrate reductase enzymes that catalyze the reduction in nitrates to nitrites within the gastrointestinal tract [45,46].

#### 3.2.6. Indole Derivatives

Tryptophan is an essential amino acid that converts to indole derivatives (indole-3-acetic acid, indole-3-sulfate, indole-3-propionic acid, and indole-3-aldehyde, etc.) under the action of tryptophanase. Tryptophan and its derivatives are considered important biomarkers and therapeutic targets, as they have anti-inflammatory properties, thus having a protective role in CRC development through an aryl hydrocarbon receptor-dependent manner. While the human body cannot synthesize tryptophan, some gut bacteria can produce it and transform it into its derivatives. Modulation of microbiota through the administration of several Lactobacillus strains, *Lactobacillus gallinarum*, *Lactobacillus rhamnosus* (*L. rhamnosus*), and *Lactobacillus reuteri*, is thought to suppress intestinal tumor growth by increasing beneficial bacteria, reducing pathogenic CRC pathogens, and promoting apoptosis [47,48].

#### 3.2.7. Trimethylamine *N*-Oxide

TMAO has been extensively studied, particularly in the cardiovascular field, where it has been proposed as a potential prognostic biomarker [49]. Higher levels of TMAO have been associated with inflammatory responses that may contribute to colorectal carcinogenesis. A recent preclinical study suggested that TMAO may promote intestinal carcinogenesis by inhibiting the FXR–FGF15 signaling pathway and activating Wnt/β-catenin signaling [50]. However, these findings require validation in large-scale prospective clinical studies, and whether TMAO plays a causal role in colorectal carcinogenesis or merely reflects disease-related metabolic alterations remains unclear. This uncertainty is reflected in inconsistent clinical literature, with some studies demonstrating no prognostic or diagnostic value for TMAO [51]. Ultimately, these conflicting data limit the clinical utility of TMAO as a dependable biomarker at this stage.

#### 3.2.8. Polyamines

The main polyamines found in the intestine are putrescine, spermine, spermidine, and cadaverine [52]. They are obtained either from the diet, synthesized by the host, or certain bacteria and are involved in various biological processes. When found in physiological concentrations, they have anti-inflammatory properties by decreasing lipopolysaccharide (LPS)-induced IL-1 and interferon-γ and upregulating interleukin-10 synthesis. They also maintain the integrity of the intestinal wall by expression of Toll-like receptor 2 (TLR2), secretion of mucin, and induction of E-cadherin. However, high concentrations of polyamines, especially *N*^1^,*N*^12^-diacetylspermine, lead to increased ROS, thus DNA damage, which contributes to CRC development. There seems to be a synergistic relationship between the tumor and the bacteria in the formed biofilm. Tumor cells produce polyamines that generate bacterial biofilms, which in turn produce more polyamines for the cancer tissue (Table 1) [53,54,55].

### 3.3. Host-Microbiota Interactions in CRC: TLR Signaling and Bacterial Extracellular Vesicles

#### 3.3.1. Toll-like Receptors (TLR)

Toll-like receptors are a type of pattern-recognition receptor that is part of the innate immune system, which plays an important role in recognizing pathogen-associated molecular patterns (PAMPs) and damage-associated molecular patterns (DAMPs). There are 10 known TLRs, but the most studied in CRC development are TLR2, TLR4, TLR5, and TLR 9. The main PAMPs found in gut bacteria composition are LPS, peptidoglycan, flagellin, and cytosine–phosphate–guanine DNA. Following ligand binding, TLRs initiate intracellular signaling pathways through myeloid differentiation response 88, TIR domain-containing adaptor protein, TIR-domain-containing adapter-inducing interferon-β (TRIF), and TRIF-related adaptor molecule, leading to activation of NF-κB, p38 mitogen-activated protein kinase, JNK, and interferon signaling, which ultimately induces cytokine production and innate immune responses. Furthermore, overstimulation contributes to tumor cell proliferation and survival. Considering their dual role, TLRs can also contribute to the modulation of anti-tumor immune response [56,57,58,59].

#### 3.3.2. Bacterial Extracellular Vesicles (BEVs)

Bacterial extracellular vesicles are nanosized membrane particles that contain nucleic acids, lipids, proteins, and virulence factors. These vesicles are released by gut bacteria and are thought to be the link between bacteria and host cells due to their ability to pass through biological barriers without causing adverse reactions. Consequently, they represent promising candidates both as biomarkers and as bioengineered vehicles for drug delivery [60].

### 3.4. Bacteria Involved in CRC Development

The gut microbiota is crucial in maintaining homeostasis, preserving the barrier function, and preventing colonization by pathogenic bacteria. Dysbiosis contributes to carcinogenesis by promoting chronic inflammation, altered dietary metabolites, genotoxins, and immune dysregulations [61,62]. Some bacteria are increased in patients with CRC, while others, with protective roles, decrease. The most studied bacteria involved in colorectal tumorigenesis are *F. nucleatum*, Polyketide synthase+ (pks+) *E. coli*, ETBF, *E. faecalis*, *Streptococcus bovis/gallolyticus*, *Helicobacter pylori*, *Clostridium septicum* (*C. septicum*), and *Peptostreptococcus anaerobius* (*P. anaerobius*). Lactobacillus strains and Bifidobacterium, on the other hand, exert anti-inflammatory effects [63].

### 3.5. Fusobacterium nucleatum

*F. nucleatum* is a Gram-negative bacterium found in oral cavities, often linked with periodontal disease, cardiovascular diseases, inflammatory bowel disease (IBD), and CRC [64]. Its presence is associated with unfavorable prognostics in patients with colorectal cancer [65]. It is still unclear whether *F. nucleatum* is a promoter of CRC, a passenger bacterium, or just an opportunistic colonizer [66,67].

*F. nucleatum* produces FadA adhesin that, by binding to E-cadherin, activates the β-catenin pathway, leading to increased transcription of oncogenes and enhanced cellular proliferation [68,69]. Additionally, *F. bacterium* has an outer membrane protein, Fap2, which interacts with the T cell immunoglobulin and ITIM domain receptor on natural killer cells and T lymphocytes, manifesting its capacity to evade host immune responses by impairing the immune cell activity [64,70]. Chronic inflammation is another key mechanism linking *F. nucleatum* to CRC. By activating the NF-κB pathway, it triggers the production of cytokines (IL-6, interleukin-8 (IL-8), interleukin 1β, and TNF-α, etc.), leading to cell proliferation and inhibition of apoptosis [71]. It further promotes genomic instability, thus DNA damage, and modulates the tumor microenvironment [72,73]. *F. nucleatum* has been found even in CRC distant metastases and is linked to resistance to chemotherapy by downregulating the expression of miR-4802 and miR-18a [63,70,74]. A very recent study described a novel circPTP3/miR-760/PUM1 regulatory axis through which *F. nucleatum* promotes CRC progression, making circPTP3 a biomarker candidate and therapeutic target [75].

### 3.6. Escherichia coli

*E. coli* is a Gram-negative bacillus commonly found in the human intestine. However, there are 4 important strains of *E. coli*: A, B1, B2, and D. A and B1 strains are commensal, while B2 and D are pathogenic. Pathogenic strains possess virulence factors such as cycle inhibiting factor (CIF), cytolethal distending toxins, cytotoxic necrotizing factor, and colibactin. The most studied strain is polyketide synthase+ (pks+) *E. coli* from group B2, which synthesizes colibactin, a genotoxin that causes DNA double-strand breaks and chromosomal instability. It further disrupts the gut epithelial barrier, promotes a pro-inflammatory microenvironment, and modulates the host signaling pathways. Consequently, *E. coli* has been recognized as a “driver” bacterium in CRC development [76,77,78,79,80,81,82] and even proposed as an adenoma-associated biomarker [80]. It has also been discovered that pks+ *E. coli* could interfere with the efficiency of chemotherapy. By decreasing cytotoxic T-cell levels and suppressing immune cell invasion, it hinders the potential of anti-programmed cell death protein 1 (PD-1) drugs [76,83].

Several approaches have been explored to reduce colibactin production and its harmful effects. Strategies to combat colibactin production include inhibiting key enzymes involved in its activation, such as polyphosphate kinase using 5-aminosalicylic acid, and colibactin peptidase using boron-based compounds. Other strategies involve modulating metabolic factors like iron and spermidine and using natural compounds that downregulate *clb* gene expression (cinnamon derivatives, plant extracts, tannins, and quercetin). Dietary interventions (e.g., green tea) and microbiota-based approaches, including prebiotics and probiotics, are also being explored, although further validation is needed [83].

### 3.7. Bacteroides fragilis

*B. fragilis* is a Gram-negative bacterium subdivided into enterotoxigenic *Bacteroides fragilis* and nontoxigenic *Bacteroides fragilis* (NTBF). ETBF can cause acute diarrhea but has also been associated with IBD and CRC. ETBF possesses a virulence factor known as *B. fragilis* toxin (BFT), also called fragilysin, which causes e-cadherin cleavage, thus activating the wingless-related integration site (Wnt) signaling pathway that leads to disruption of the epithelial barrier, increased cell proliferation, and reduced apoptosis. Moreover, by binding to the colonic epithelial cell receptor, it activates the STAT3 pathway through IL-17 and IL-8 pathways, leading to the production of ROS and DNA damage [84,85,86,87,88]. Another mechanism of promoting tumorigenesis is through TLR4 by developing stem-like properties in colorectal epithelial cells. BF’s ability to form biofilms amplifies its oncogenic potential by promoting persistent epithelial interaction, chronic inflammation, and immune evasion, creating a tumor-permissive microenvironment. Its presence in mucosal biopsies from patients with precancerous lesions and low-grade dysplasia supports the classification of ETBF as a “driver” bacterium, making it a valuable biomarker for early CRC detection [89,90].

### 3.8. Enterococcus faecalis

*E. faecalis* is a Gram-positive bacterium implicated in colorectal cancer development through its capacity to induce direct DNA damage. It generates ROS, particularly extracellular superoxide, which causes oxidative DNA damage. This process leads to chromosomal instability, accumulation of mutations, and disruption of normal cell cycle regulation, thereby contributing to tumor initiation and progression [91,92,93,94,95]. A recent study showed that *E. faecalis* could promote CRC progression by producing biliverdin, which activates pathways like PI3K/AKT/mTOR. This enhances tumor cell proliferation and angiogenesis, although current evidence remains limited and requires further validation [96].

### 3.9. S. gallolyticus subsp. gallolyticus

*S. gallolyticus* is a Gram-positive bacterium that corresponds to biotype I within the *Streptococcus bovis-Streptococcus equinus complex* [97]. Endocarditis and bacteremia caused by this opportunistic pathogen appear to be an indicator for performing colonoscopy, as it is strongly associated with colorectal neoplasia. *S. gallolyticus* stimulates cell proliferation by activating the Wnt/β-catenin signaling pathway and promotes a pro-inflammatory environment by overexpressing cyclooxygenase-2, being considered an early biomarker in CRC detection, or even a marker for the risk of developing CRC [98,99,100,101,102]. However, more research is needed in order to elucidate the exact pathogenic mechanism and link to CRC tumorigenesis.

### 3.10. Peptostreptococcus anaerobius

The effects of *P. anaerobius* are driven by its interaction with TLR2 and TLR4 on host cells, which leads to increased ROS production, upregulation of cholesterol biosynthesis through modulation of sterol regulatory element-binding protein 2, and activation of pro-oncogenic pathways that contribute to the development of colorectal cancer [95]. *P. anaerobius* adheres preferentially to colorectal cancer cells, activating the NF-κB signaling pathway by binding PCWBR2-integrin α2β1, leading to cell proliferation and inflammation. It further inhibits ferroptosis and promotes tumorigenesis by influencing the tumor immune microenvironment through myeloid-derived suppressor cells (MDSCs). Suppressing MDSCs leads to anti-PD1 therapy resistance [103,104].

### 3.11. Clostridium septicum

*C. septicum* is an anaerobic bacterium of ongoing research interest due to its association with CRC. Previously, it was shown that patients with *C. septicum* bacteremia were correlated with a higher incidence of colorectal malignancy, supporting the hypothesis that this bacterium is an opportunistic pathogen that exploits the tumor microenvironment of pre-existing CRC [105]. A recent study raised the supposition that *C. septicum* could actively contribute to the process of carcinogenesis through DNA damage, but there is no clear evidence to support this [106]. However, *C. septicum* remains a biomarker candidate for advanced CRC [100,107].

### 3.12. Helicobacter pylori

*H. pylori*’s role in CRC development is controversial, as contradictory study results exist. *H. pylori* is thought to promote CRC development indirectly. A recent meta-analysis points out a strong association between *H. pylori* and colorectal cancer and adenoma, revealing the impact of *H. pylori* eradication [108]. Ralser et al. describe the mechanisms of carcinogenesis by infecting the stomachs of *Apc*^+/min^ and *Apc*^+/1638N^ mice, concluding that *H. pylori* promotes a pro-inflammatory environment and produces gut microbiome dysbiosis, leading to epithelial barrier disruption [109]. Furthermore, elevated levels of gastrin stimulate colonocyte proliferation [108]. However, extensive research is needed to establish the exact implication of *H. pylori* in CRC development.

Among CRC-associated bacteria, *F. nucleatum* is one of the most extensively studied candidates for translational biomarker development, supported by consistent enrichment in colorectal tumor tissue and stool samples, as well as associations with disease progression. However, no bacterial species has yet achieved clinical readiness or routine use in CRC screening (Table 2).

### 3.13. Protective Bacteria

**Figure 3:**
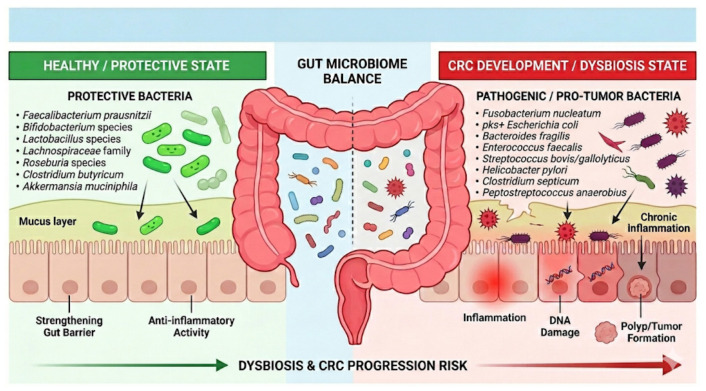
Bacteria that are linked to CRC development and protective bacteria. Abbreviations: DNA, deoxyribonucleic acid; pks^+^, polyketide synthase positive.

The gut microbiota of colorectal cancer patients consists of an increase in the bacteria mentioned before and a decrease in beneficial bacteria like *Faecalibacterium prausnitzii* (*F. prausnitzii*), Bifidobacterium species, Lactobacillus species, Lachnospiraceae family, Roseburia species, *Clostridium butyricum* (*C. butyricum*), *Akkermansia muciniphila,* and others [97,111]. Some of them exert a protective role by producing butyrate (*F. prausnitzii,* Roseburia, *Eubacterium rectale*, Coprococcus, and *C. butyricum*) or indole-3-lactic acid (*Lactobacillus plantarum*), while others modulate the immune response (Lachnospiraceae), enhance PD-1/PD-L1 checkpoint efficacy (*Lactobacillus rhamnosus*, *Lactobacillus paracasei* (*L. paracasei*), Bifidobacterium species, and *F. prausnitzii*), produce anti-inflammatory metabolites like picolinic acid, conjugated linoleic acid and contribute to maintaining the epithelial barrier integrity and mucus layer (Figure 3) [111,112,113,114,115,116].

## 4. Sporadic CRC vs. IBD-Associated CRC

CRC develops through two main pathways: sporadic CRC and inflammatory bowel disease-associated CRC. Although both lead to malignant transformation of the colonic epithelium, they differ markedly in initiating factors and underlying mechanisms. Sporadic CRC follows the classical adenoma-carcinoma sequence, characterized by progressive accumulation of genetic mutations such as APC, KRAS, and TP53 [117,118]. In this setting, microbiota acts mainly as a modulator of tumor progression [119]. IBD-associated CRC develops through an inflammation-dysplasia-carcinoma sequence driven by persistent mucosal inflammation. Chronic immune activation leads to epithelial injury, oxidative stress, and regenerative proliferation, promoting neoplastic transformation. In this context, microbial dysbiosis is more central, characterized by reduced diversity and expansion of pathobionts that perpetuate inflammation and tumorigenesis [120,121]. Despite distinct origins, both CRC types converge on common mechanisms, including epithelial barrier disruption, chronic immune activation, and microbiota-driven metabolic changes. In both, microbial metabolites and toxins contribute to tumor-promoting signaling, supporting the concept of an inflammation-microbiota-tumor axis in colorectal carcinogenesis (Table 3) [122,123].

## 5. Gut Microbiota Modulation

The research for gut microbiota modulation is ongoing, with various strategies being proposed and tested, such as dietary intervention, administration of selective antibiotics, probiotics, prebiotics, synbiotics, postbiotics, bacteriophages, and FMT (fecal microbiota transplantation) [125].

Antibiotics have shown promising results in overcoming chemotherapeutic resistance and targeting certain harmful bacteria, but broad-spectrum antibiotics often lead to dysbiosis and antimicrobial resistance [90,126]. Metronidazole and therapeutic combinations with metronidazole decrease F. bacterium levels and reduce the progression of colorectal cancer [127].

Administration of probiotic strains has promising results in reducing the CRC risk and progression. Most studied strains are *L. rhamnosus*, *Bifidobacterium longum*, *L. casei*, *L. paracasei*, *Lactobacillus reuteri*, *C. butyricum*, *Lactobacillus plantarum*, *Butyricicoccus pullicaecorum*, *Propionibacterium freudenreichii*, *Lactobacillus acidophilus*, *Bifidobacterium infants*, *Bifidobacterium breve,* and *Lactobacillus gallinarum*, which in various combinations have shown good results [47,125,128,129,130,131,132].

Prebiotics (inulin, fructo-oligosaccharides, galacto-oligosaccharides) modify the gut microbiota positively by stimulating beneficial bacterial growth and SCFA production [133,134]. Berberine has a prebiotic-like effect by modulating the gut microbiota, decreasing inflammation, and suppressing tumor growth [135,136,137].

Postbiotics are considered a safe adjuvant treatment for CRC. The principal components of postbiotics comprise inactivated microbial cells, bacterial fractions (including cytosolic polypeptides, phosphoglycolic acids, intracellular and extracellular polysaccharides), and surface-associated proteins, as well as their metabolites, such as SCFAs, organic acids, bacteriocins, and enzymes [138].

*Bacteriophages* are an emerging therapy using nanotechnology for targeted microbiota modulation, without affecting the beneficial bacteria. However, further studies and clinical trials are required before effective prototypes can be developed and implemented [125,126].

*FMT* is an emerging strategy of modulating gut microbiota with considerable therapeutic potential. With extraordinary effects against *Clostridium difficile* infection, further studies showed beneficial results in obesity, insulin resistance, metabolic syndrome, chronic fatigue syndrome, and fibromyalgia, as well as neurological and neurodevelopmental disorders including Parkinson’s disease, multiple sclerosis, myoclonus–dystonia, and autism [139]. In the gastroenterology field, FMT has shown encouraging outcomes in IBD, constipation [119,140], and colorectal cancer treatment. The principal roles observed in CRC patients include enhancing therapeutic efficacy and minimizing therapy’s adverse effects on healthy tissues [141,142,143]. However, FMT is still controversial as it poses a high risk of transmitting various diseases. Rigorous donor selection and screening must be performed with close monitoring and standardized administration protocols established.

## 6. Early Gut Microbiota Signatures

In search of potential gut microbiota bacterial biomarkers, it has been found that various bacterial strains are increased in patients with adenoma or early colorectal cancer. According to a study performed by Zhang et al., in the adenoma–carcinoma shift, there was an increase in *Peptostreptococcus stomatis*, *Parvimonas micra*, *Gemella morbillorum*, *Dialister pneumosintes*, *Porphyromonas asaccharolytica*, *Solobacterium moorei*, *Eisenbergiella tayi*, *F. nucleatum*, *Ruminococcus torques*, *Eggerthella lenta*, *Clostridium symbiosum*, *Campylobacter rectus*, *Clostridium scindens,* and *Clostridium lactatifermentans* in stool samples [144]. Another study concluded that *Bacteroides ovatus* is increased in patients progressing from adenomas to CRC and might be a potential early fecal biomarker, while *Streptococcus thermophilus*, *Eubacterium ventriosum*, Streptococcus, Erysipelotrichaceae, *Coprococcus* sp_ART55_1, and *Eubacterium siraeum* protect against CRC [145]. A review performed by Zhou et al. concluded that Bifidobacteria, *F. nucleatum*, *Geotrichum candidum*, *Porphyromonas asaccharolytica*, *E. coli*, Rhodococcus, *Anaerostipes caccae*, Enhydrobacter, *Lachnoclostridium* sp.m3, Bacteroides clarus, *Clostridium hathewayi*, Ruminococcaceae, *Bacteroides thetaiotaomicron*, Culinariside, and ETBF could be efficient early biomarkers [146]. Zhang et al. found an increase in *Ruminococcus gnavus*, *Bacteroides ovatus*, *Parabacteroides distasonis*, *Citrobacter freundii*, and *Clostridium symbiosum* in patients with colorectal polyps [147].

Even though there is no consensus, several combinations of strains combined with preexistent tests for early colorectal cancer have been proposed. The combination of *F. nucleatum*, Lachnoclostridium species, *Clostridium hathewayi*, *Bacteroides clarus,* and fecal immunochemical test (FIT) has achieved a specificity of 81.2% and a sensitivity of 93.8% in CRC detection [148]. Moreover, the combination of 28 operational taxonomic units from oral and stool microbiota reached a specificity of 88% and a sensitivity of 94% [149].

## 7. Conclusions and Future Directions

In conclusion, gut microbiota-derived metabolites exert both protective and harmful effects in CRC. SCFAs, particularly butyrate, show anti-inflammatory and antitumor properties, whereas metabolites such as deoxycholic acid, lithocholic acid, hydrogen sulfide, and nitroso compounds are linked to carcinogenesis. TMAO exhibits potential pro-inflammatory and pro-tumorigenic effects, while 3-oxo-LCA contrasts this by exerting immunomodulatory properties that support anti-inflammatory pathways. Indoles generally promote anti-inflammatory responses, while other tryptophan metabolites may enhance inflammation. Similarly, polyamines display a dual role in tumorigenesis, with their impact largely dependent on concentration and metabolic context.

Apart from the very well-known strains found in CRC patients (*F. nucleatum*, Pks+ *E. coli*, *B. fragilis*, *E. faecalis*, *S. bovis/gallolyticus*, *H. pylori*, *C. septicum,* and *P. anaerobius*), there is a big inconsistency in the strains found in the gut microbiota of patients with polyps, adenoma, or early carcinoma. Although these microbial biomarkers hold promise for enhancing early detection and risk stratification in colorectal cancer, no bacterial marker has yet reached the level of validation required for standardized clinical use.

The main obstacle in implementing standardized early biomarkers is due to variations in gut microbiota with diet, geography, and other pathologies that present with altered microbiota, topped by the lack of standardization of sample collecting methods and processing, the use of different DNA extraction kits, and different sequencing methods in studies. There is an acute need for long-term multicentered standardized Randomized Controlled Trials around the globe, with clear protocols, to truly understand the mechanisms of action of bacteria in colon tumorigenesis and the succession in polyp-adenoma-CRC development, alongside blood, saliva, fecal, and tumor samples collected from the same patient. Moreover, our current understanding of the exact role of certain increased bacteria from gut microbiota is limited. It is still not clear if the bacteria are casually there, if they opportunistically populate the site of cancer, or if they truly influence the tumorigenesis process. What seems to be clear is that the specificity and sensitivity of colorectal cancer detection are better when combining oral and fecal microbiome biomarkers in addition to preexistent tests, like FIT.

The main objective is to develop efficient, non-invasive screening strategies that surpass current methods for the early detection of colorectal cancer. Further research should provide important insights for the targeted modulation of the gut microbiota, both as a preventive approach against colorectal cancer development in high-risk individuals and as a potential adjuvant strategy in colorectal cancer therapy. In this context, artificial intelligence and deep learning approaches could help overcome current limitations by integrating heterogeneous microbiome datasets from multiple populations and platforms, identifying robust microbial signatures despite technical and biological variability, and improving the predictive accuracy of multi-omics models for early colorectal cancer detection and risk stratification.

## Figures and Tables

**Table 1:** Main metabolites that influence CRC development.

Metabolite	Pathway	Source	Category	References
Butyrate (SCFA)	Histone deacetylase inhibition, apoptosis of tumor cells, modulates the immune system, maintains intestinal barrier integrity	Dietary fibers	Strongly protective	[16,32,33]
Acetate (SCFA)	Supports epithelial energy balance	Dietary fibers	Protective	[35]
Propionate (SCFA)	Anti-inflammatory properties, induces cell apoptosis and inhibits cholesterol synthesis	Dietary fibers	Protective	[36]
Deoxycholic acid	Activates NF-κB pathway, DNA damage, disrupts intestinal barrier integrity, chronic inflammation	High fat diets	Harmful	[33,37]
Lithocholic acid	Activates ERK1/2 and inhibits STAT3	High fat diets	Harmful	[38,39,40,41]
Hydrogen sulfide (H_2_S)	Genotoxicity and epithelial hyperproliferation	Processed/red meats	Harmful	[43,44]
Nitroso compounds (NOCs)	DNA alkylation and mutations	Processed meats	Harmful	[45,46]
Indole derivatives	AhR activation, increase beneficial bacteria, reduce pathogenic bacteria, promote apoptosis, anti-inflammatory properties	Tryptophan-rich foods	Protective	[47,48]
Trimethylamine *N*-oxide	Promotes inflammation and inhibits FXR-FGF15	Dietary choline, L-carnitine and hepatic	Potentially harmful	[49,50,51]
Polyamines	DNA damage	Diet, synthesized by host or certain bacteria	Harmful in excess	[53,54,55]

Abbreviations: AhR, aryl hydrocarbon receptor; DNA, deoxyribonucleic acid; ERK1/2, extracellular signal-regulated kinases 1 and 2; FGF15, fibroblast growth factor 15; FXR, farnesoid X receptor; H_2_S, hydrogen sulfide; NF-κB, nuclear factor kappa B; NOCs, *N*-nitroso compounds; SCFA, short-chain fatty acid; STAT3, signal transducer and activator of transcription 3.

**Table 2:** The most studied bacteria implicated in CRC development, their pathways, early detection evidence, and clinical relevance.

Bacteria	Main Pathway/Mechanism in CRC	Early Detection Evidence	Clinical Relevance	References
*Fusobacterium nucleatum*	FadA → E-cadherin/β-catenin activation; Fap2 → TIGIT-mediated immune evasion; NF-κB–mediated inflammation; miRNA modulation and circPTBP3/miR-760/PUM1 axis	Strong association in CRC tissue and stool; correlates with tumor stage	Promising non-invasive biomarker-stool based tests under development	[28,63,64,65,66,67,68,69,70,71,72,73,75]
*Escherichia coli* (pks^+^)	Colibactin production → DNA double-strand breaks, chromosomal instability, inflammation, immune suppression	Moderate microbiome association in CRC cohorts	Early-stage biomarker candidate	[76,77,78,79,80,81,82,83,110]
*Bacteroides fragilis*	BFT → E-cadherin cleavage; Wnt activation; IL-17/STAT3 signaling; ROS-mediated DNA damage	Moderate CRC microbiome association	Exploratory biomarker	[84,85,86,87,88,89,90]
*Enterococcus faecalis*	ROS generation; oxidative DNA damage; PI3K/AKT/mTOR activation via biliverdin	Weak–moderate association in CRC microbiome studies	Investigational biomarker	[91,92,93,94,95,96]
*S. gallolyticus* subsp. *gallolyticus*	Wnt/β-catenin activation; COX-2 overexpression; inflammation and proliferation	Strong clinical association (CRC-related bacteremia/endocarditis)	Clinical warning marker	[98,99,100]
*Peptostreptococcus anaerobius*	TLR2/TLR4 signaling; ROS production; SREBP2-mediated cholesterol biosynthesis; NF-κB activation; ferroptosis inhibition	Emerging CRC-associated microbial signature	Emerging biomarker	[95,103,104]
*Clostridium septicum*	Association with CRC microenvironment; possible contribution to DNA damage and carcinogenesis	Moderate clinical association via bacteremia	Clinical red flag organism, candidate investigational biomarker	[100,105,106,107]
*Helicobacter pylori*	Dysbiosis; epithelial barrier disruption; gastrin-mediated proliferation; inflammation	Inconsistent CRC association	Low specificity for CRC detection	[108,109]

Abbreviations: AKT, protein kinase B; BFT, *Bacteroides fragilis* toxin (fragilysin); circPTBP3, circular RNA polypyrimidine tract-binding protein 3; COX-2, cyclooxygenase-2; CRC, colorectal cancer; DNA, deoxyribonucleic acid; E-cadherin, epithelial cadherin; FadA, *Fusobacterium* adhesin A; Fap2, *Fusobacterium* autotransporter protein 2; IL-17, interleukin-17; miR-760, microRNA-760; miRNA, microRNA; mTOR, mammalian (mechanistic) target of rapamycin; NF-κB, nuclear factor kappa B; PI3K, phosphoinositide 3-kinase; pks^+^, polyketide synthase positive; PUM1, Pumilio RNA-binding family member 1; ROS, reactive oxygen species; SREBP2, sterol regulatory element-binding protein 2; STAT3, signal transducer and activator of transcription 3; TIGIT, T-cell immunoreceptor with immunoglobulin and ITIM domains; TLR2, Toll-like receptor 2; TLR4, Toll-like receptor 4; Wnt, Wingless/Integrated signaling pathway.

**Table 3:** Mechanistic and pathogenetic distinctions between sporadic and IBD-associated colorectal cancer.

Feature	Sporadic CRC	IBD-Associated CRC
Primary initiating factor	Accumulation of genetic mutations (APC, KRAS, TP53) [118]	Chronic intestinal inflammation [121]
Carcinogenic sequence	Adenoma → carcinoma sequence [118]	Inflammation → dysplasia → carcinoma [121]
Role of inflammation	Secondary/modulatory [124]	Central driver [120]
Microbiota role	Contributor to progression and tumor modulation [122]	Key initiator and amplifier of inflammation [120]
Microbial changes	Enrichment of oncogenic bacteria (e.g., *F. nucleatum*) [119]	Reduced diversity, expansion of pathobionts [120]
Key mechanisms	DNA damage, immune evasion, signaling activation [119]	Oxidative stress, epithelial injury, chronic immune activation [117]

Abbreviations: CRC, colorectal cancer; IBD, inflammatory bowel disease; APC, adenomatous polyposis coli gene; KRAS, Kirsten rat sarcoma viral oncogene homolog; TP53, tumor protein p53; *F. nucleatum*, *Fusobacterium nucleatum*.

## Data Availability

All data are contained within the article.
